# The medicinal activity of lyophilized aqueous seed extract of *Lepidium sativum L.* in an androgenic alopecia model

**DOI:** 10.1038/s41598-023-33988-1

**Published:** 2023-05-11

**Authors:** Marzough Aziz Albalawi, Ahmed M. Hafez, Seham S. Elhawary, Nada K. Sedky, Omnia F. Hassan, Rofanda M. Bakeer, Soha Abd El Hadi, Ahmed H. El-Desoky, Sebaey Mahgoub, Fatma A. Mokhtar

**Affiliations:** 1 Department of Chemistry, Alwajh College, University of Tabuk, Tabuk, Saudi Arabia; 2 Department of Biochemistry, School of Life and Medical Sciences, University of Hertfordshire Hosted by Global Academic Foundation, Cairo, Egypt; 3 Department of Pharmacognosy, Faculty of Pharmacy, Cairo University, Cairo, Egypt; 4 Department of Pharmacology and Toxicology, Faculty of Pharmacy, MSA University, 6th of October City, Egypt; 5 Department of Pathology, Faculty of Medicine, Helwan University, Helwan, Egypt; 6 Department of Pharmaceutical Chemistry, Faculty of Pharmacy, Egyptian Russian University, Badr City, Cairo, Egypt; 7 Department of Pharmacognosy, Pharmaceutical Industries Research Institute, National Research Centre, Dokki, Giza, 112611 Egypt; 8 Food Analysis Laboratory, Ministry of Health, Zagazig, 44511 Egypt; 9 Department of Pharmacognosy, Faculty of Pharmacy, El Saleheya El Gadida University, El Saleheya El Gadida, Sia, 44813 Egypt

**Keywords:** Biochemistry, Chemical biology, Drug discovery, Medical research, Molecular medicine

## Abstract

This study evaluated the topical effect of *Lepidium sativum* lyophilized seed extract (LSLE) towards Sustanon-induced alopecia in male adult Wistar albino rats in vivo, compared to minoxidil topical reference standard drug (MRD). LC–MS/MS together with molecular networking was used to profile the metabolites of LSLE. LSLE treated group revealed significant changes in alopecia related biomarkers, perturbation of androgenic markers; decline in testosterone level and elevation in 5α-reductase (5-AR); decline in the cholesterol level. On the other hand, LSLE treated group showed improvement in vascular markers; CTGF, FGF and VEGF. Groups treated topically with minoxidil and LSLE showed significant improvement in hair length. LC–MS/MS profile of LSLE tentatively identified 17 constituents: mainly glucosinolates, flavonoid glycosides, alkaloids and phenolic acids. The results point to the potential role of LSLE in the treatment of alopecia through decreasing 5(alpha)-dihydrotestosterone levels. Molecular docking was attempted to evaluate the probable binding mode of identified compounds to androgen receptor (PDB code: 4K7A).

## Introduction

Alopecia is a dermatological illness that has been known for over a thousand years and it is a common concern in both cosmetic and basic health care. Androgenic alopecia (AGA) is considered the most prevalent type of hair loss and is commonly used to define the condition of scalp hair loss in both males and females who are genetically susceptible to it^1^. It is characterized by the increased activity of the 5α-reductase (5AR) enzyme which accelerates the reduction of testosterone to 5α-dihydrotestosterone (DHT). Despite the fact that androgens are responsible for some secondary sexual characteristics such as facial hair growth, DHT miniaturizes androgen-sensitive follicles in the scalp causing thinning of the scalp hair^2^. Meanwhile, oxidative stress was found to accelerate cell senescence of dermal papilla cells via stimulating of the apoptotic pathways within hair follicles, resulting in faster hair loss in AGA. Thus, antioxidants could be regarded as a beneficial auxiliary treatment to hinder hair loss speed in AGA^3^.

Finasteride and minoxidil are both FDA-approved to treat AGA. Finasteride inhibits the 5α-reductase activity while minoxidil can enhance hair growth via vasodilatation caused by opening potassium channels located on smooth muscle cells of peripheral arteries with gradual slowing of circulation. Minoxidil topical solution is a clinically approved and effective hair growth stimulant. It can also reduce hair loss and maintain hair growth^4^.

Several Brassicaceae plants have been reported to promote hair growth in AGA treatment such as *Brassica oleracea* extract, its glucosinolate rich fraction^5^, wasabi derived 6-methylsulfinylhexyl isothiocyanate^6^ and watercress extract^7^. Meanwhile, *Lepidium sativum* also known as garden cress exhibited antiandrogenic activity^8^. Therefore, Brassicaceae plants are regarded as potential candidates for the treatment of AGA due to their contents of glucosinolates^5–8^ and potential antioxidant phenolic compounds, such as phenolic acids and flavonoids^9^. Meanwhile, *Lepidium sativum* exhibited other potentials anti-inflammatory^8^ and antioxidant effects^10,11^.

In the current study, Sustanon, which is a mixture of propionate, phenyl propionate, isocaproate and decanoate esters of testosterone was used to induce AGA. The current study sought to investigate the potential use of LSLE as a treatment for androgenic alopecia and compare the results to the market's standard 5% minoxidil.

## Materials and methods

### Preparation of *Lepidium sativum* L. lyophilized seed extract (LSLE)

The *Lepidium sativum* L. dry seeds were purchased from the Egyptian local market as they are edible seeds and authenticated by Prof. Amal Fakhry at Faculty of Science, Alexandria University, Egypt. A voucher specimen (PG-A-SD-F-16) was deposited at the plant department, Faculty of Science Tanta University. The seeds (2 kg) were coarse ground and soaked in distilled H_2_O (3 × 7L) at room temperature with frequent shaking for 12 h. The soaked seeds were cold pressed, filtered, and the filtrate was centrifuged at 3000 rpm for 15 min. The supernatant was separated in a gel form and lyophilized. After 48 h. a lyophilized dry powder was produced (140 g). The study of this species complied with relevant institutional, national, and international guidelines and legislation including the Convention on Biological Diversity and the Convention on the Trade in Endangered Species of Wild Fauna and Flora. A fraction of the LSLE was used for LC–MS/MS analysis and the rest was dissolved in normal saline for biological study.

### LC-MS/MS for metabolite profiling

Liquid chromatography-electrospray ionization–tandem mass spectrometry (LC–ESI–MS/MS) analysis was performed in the Proteomics and Metabolomics Research Program at Children’s Cancer Hospital Egypt (CCHE 57357). The sample was dissolved in a reconstitution solvent composed of water: methanol: acetonitrile (50: 25: 25 V/V), 50 mg of the sample was dissolved in 1 mL of the reconstitution solvent, vortexed for 2 min., then ultrasonicated for 10 min., centrifuged at 10,000 rpm for 10 min., then concentration was adjusted to 2.5 µg/µl before injection.

LC–MS/MS analysis was performed on Exion LC system, utilizing Xbridge C18 (3.5 µm, 2.1 × 150 mm) column (Waters Corporation, Milford, MA, USA) maintained at 40 °C. The mobile phase was freshly prepared, filtered through a membrane disc filter (0.45 μm), 10 μL of sample was injected and eluted at a 0.3 mL/min flow rate. Gradient elution of two aqueous solvents; 5 mM ammonium formate in 1% methanol adjusted to a pH of 3 using formic acid in positive ionization mode and adjusted to a pH of 8 using sodium hydroxide for negative ionization mode; and acetonitrile as the organic solvent. The gradient was programmed as follows: 10% solution B from 0 to 20 min, 90% solution B from 21 to 25 min, then 10% B for 2 min and the analytical column was finally equilibrated using 10% solution B for 1 min. The LC system was controlled and monitored by Analyst TF 1.7.1 software.

The Triple TOF 5600 + mass spectrometer (AB SCIEX, Concord, ON, Canada) with a Duo-Spray source operates in the ESI positive and negative modes. The sprayer capillary and declustering potential voltages were 4500 and 80 eV in the positive mode and − 4500 and − 80 V in the negative mode. The source temperature was 600 °C, the curtain gas pressure was 25 psi, and gas 1 and 2 pressure was 40 psi. Collision energy of 35 V (positive mode) and − 35 V (negative mode) was used with CE spreading 20 V. Spectra were recorded between 50 and 1100 m/z.

Data was processed using MS-DIAL 4.8, and the used reference databases were: ReSpect positive (2737 records) or ReSpect negative (1573 records). PeakView 2.2 with the MasterView 1.1 package were used for feature (peaks) extraction from Total ion chromatogram (TIC)^12^.

### Animals

Male adult Wistar albino rats weighing on average 200–250 g were purchased from the Egyptian Organization for Biological Products and Vaccines Egypt. The animals were caged in plexiglass cages at a temperature of 25 ± 2 °C. They were exposed to a repeated cycle of 12 h of illumination followed by 12 h of darkness. They were continuously provided with water, as well as a standard pellet diet. All the performed procedures were in alignment with the guide for the care and use of laboratory animals at the US National Institute of Health (NIH Publication No. 85-23, revised 2011). The whole experimental work was approved by the Ethics Committee for Animal Experimentation at the Faculty of Pharmacy, MSA University (PH1/EC1/2022PD) and was conducted in accordance with the ARRIVE guidelines.

### Drugs

Sustanon 250 (from Organon) and 5% minoxidil were purchased from the from a retail pharmacy and the production date checked before use.

### Induction of alopecia

At the start of the experiment part of the dorsal hair was clipped and then carefully shaved. Alopecia was then induced by subcutaneous (S.C.) injection of Sustanon dissolved in corn oil at a dose of 1 mg/kg on daily basis for a period of 21 days. The selected dose together with the utilized route of administration were adopted from literature^13,14^.

### Experimental design

**Figure 1:**
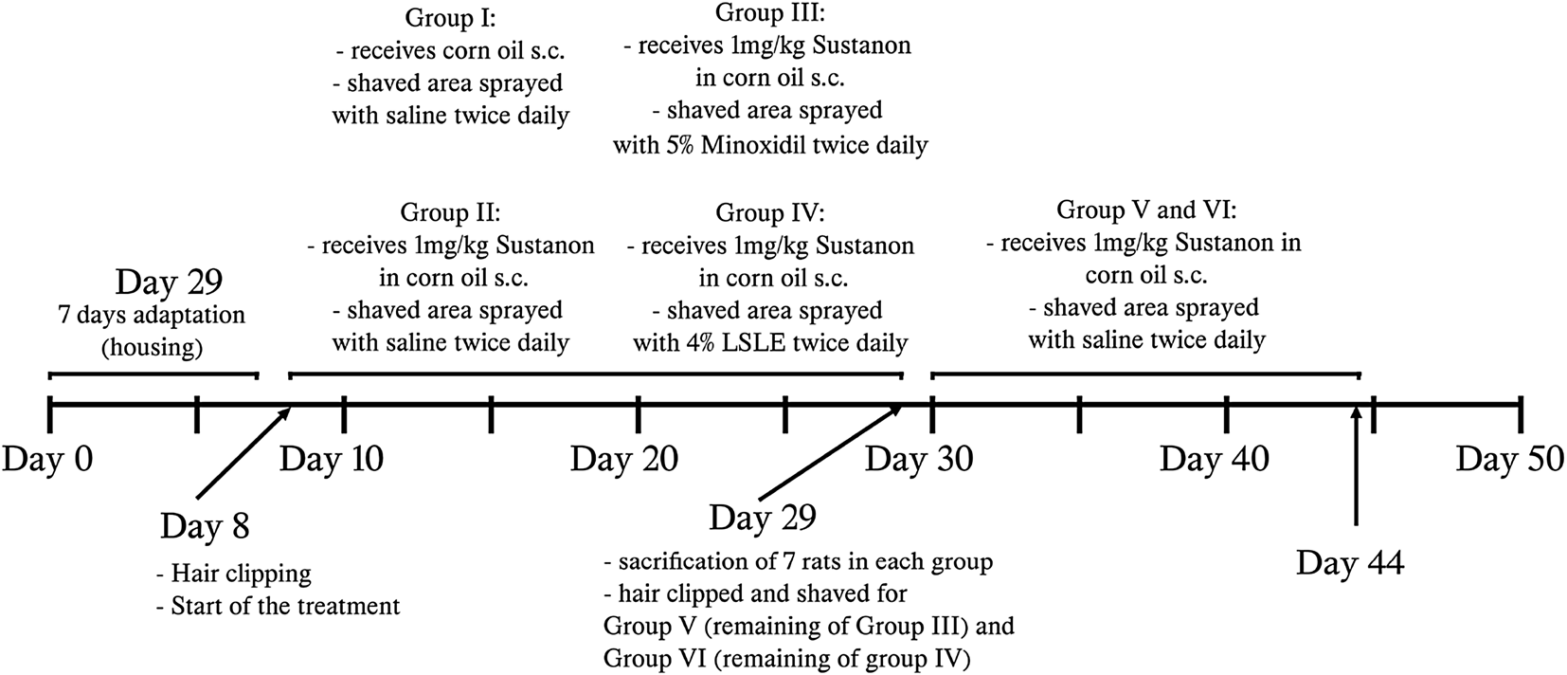
Flow chart of Experimental Design.

Forty-two male Wistar albino rats were randomly placed into four distinct groups. The rats were anesthetized then selected portions of their skin were clipped and carefully shaved with razors. Hair growth rates were observed daily. Group I (n = 7) served as a control group; rats had part of their dorsal hair clipped, shaved then received corn oil vehicle S.C. for 21 days during which their shaved portion of the skin was sprayed with saline (0.9% NaCl). The rest of the study groups had alopecia induced. Group II (n = 7) rats were sprayed with saline concurrently with Sustanon for 21 days while Group III (n = 14) had 5% minoxidil (2 sprays/rat as that was the recommended dose from the manufacturer) and Group IV (n = 14) had LSLE (4% in saline solution) (2 sprays/topically) sprayed concurrently with Sustanon for 21 days. Directly after twenty-two days, 7 rats were sacrificed from each group for skin and blood samples. The remaining 7 rats from Groups III and IV made Groups V and VI, respectively. Both groups had the topical treatment stopped and the Sustanon continued for one month to evaluate the sustained effect of the treatment (Fig. 1).

Rats were checked every 3–5 days to monitor the differing phases of hair re-growth. After 21 days, the rats were photographed and sacrificed on the following day. Blood samples were obtained, and isolated tissues were rapidly divided into 2sections. The first was fixed immediately in formalin and later used for the histopathological examination, while the latter was homogenized in phosphate-buffered saline with protease inhibitor (pH 7.4) to produce a 10% (w/v) homogenate which was suitable for the biochemical analysis.

### Biochemical assays

The blood samples were used for the measurement of cholesterol levels. A commercially available kit was used for this measurement (Biovision, USA; #K4436-100). The kit employed the sandwich ELISA principle, and the procedures were done in accordance with the manufacturer’s instructions. Cholesterol levels were finally estimated as ng/ml.

The tissue homogenate was used for the assessment of 5AR and the growth factors levels. 5AR and CTGF were assayed using sandwich ELISA from commercially available kits (Lifespan Biosciences, USA; #LS-F6847; Novus Biologicals, USA; #NBP2-75011, respectively). Both were used according to the manufacturer’s guidelines and were sensitive to the pg/ml range.

The tissue levels of fibroblast growth factor (FGF) were determined by gene expression analysis via SuperScript IV One-Step RT-PCR kit (Thermo Fisher Scientific, USA, #12594100). The forward primer was 5’ATCCTGCCGACTCCGCTCTA3’ and the reverse was 5’CCTTTTGATTTAAGGCCACGAACA3’. The 2^-ΔΔCt^ approach was used to compare the CT of each sample with that of the control group.

The Western blotting technique was used to detect the tissue levels of vascular endothelial growth factor (VEGF). After sonicating the homogenate on ice to ensure cell lysis, it was centrifuged at 4 °C for 20 min at 15,000 rpm. Protein was isolated using the Ready Prep protein extraction kit (Bio-Rad, USA; #163-2086). The total protein content was determined using the Bradford assay, and 20 μg of protein was separated on SDS-PAGE (10% polyacrylamide gel) and transferred to polyvinylidene difluoride membranes (Pierce, Rockford, IL, USA) using a Bio-Rad Trans-Blot system. The membrane was blocked for 1 h with a blocking solution (20 mM Tris–Cl (pH 7.5), 150 mM NaCl, 0.1% Tween 20, and 4% bovine serum albumin). After that, the membrane was washed twice with a wash buffer (20 mM Tris–Cl (pH 7.5) and 150 mM NaCl). The membrane was then incubated overnight at 4 °C with either the VEGF primary antibody (Abcam, UK, #ab46154) or β-actin primary antibody (Thermo Fisher Scientific Inc.) (Rockford, IL, USA). The membrane was then cleaned using wash steps. Following that, secondary antibodies labelled with horse radish peroxidase were added. The membranes were left at 25 °C for 1 h before being treated with luminol. The ChemiDoc imaging system with Image Lab software version 5.1 was used to measure the band intensity (Bio-Rad Laboratories Inc., Hercules, CA, USA). After normalization to β-actin protein levels, the results are displayed as arbitrary units.

### Histopathological assessment of hair follicles

A 4 mm punch biopsy was used for sectioning samples which were fixed in formalin 10% for 24 h, dehydrated with increasing concentrations of alcohol, then sectioned both vertically and transversely to a thickness of 5 um during the preparation. Sections were stained with H&E as well as Masson’s Trichromatic stain. All photomicrography taken was of 100 × magnification, scale bar 50 µm.

### Statistical analysis

The study used Graph Pad Prism software (version 6.04) to analyze the results. The Kolmogorov–Smirnov and Bartlett's tests were used to examine the data's normality and homogeneity of variance. The results are shown as the SD of the average of three replicates. To calculate the statistical significance between various groups, the study used one-way analysis of variance (ANOVA) followed by Tukey's post hoc test. Kruskal–Wallis non-parametric test, followed by multiple comparison Dunn's test was only used to determine the statistical significance of the hair follicle count histological score. The *P* value of ≤ 0.05 is significant throughout the manuscript.

### Molecular docking study

#### Preparation of protein receptor

**Figure 2:**
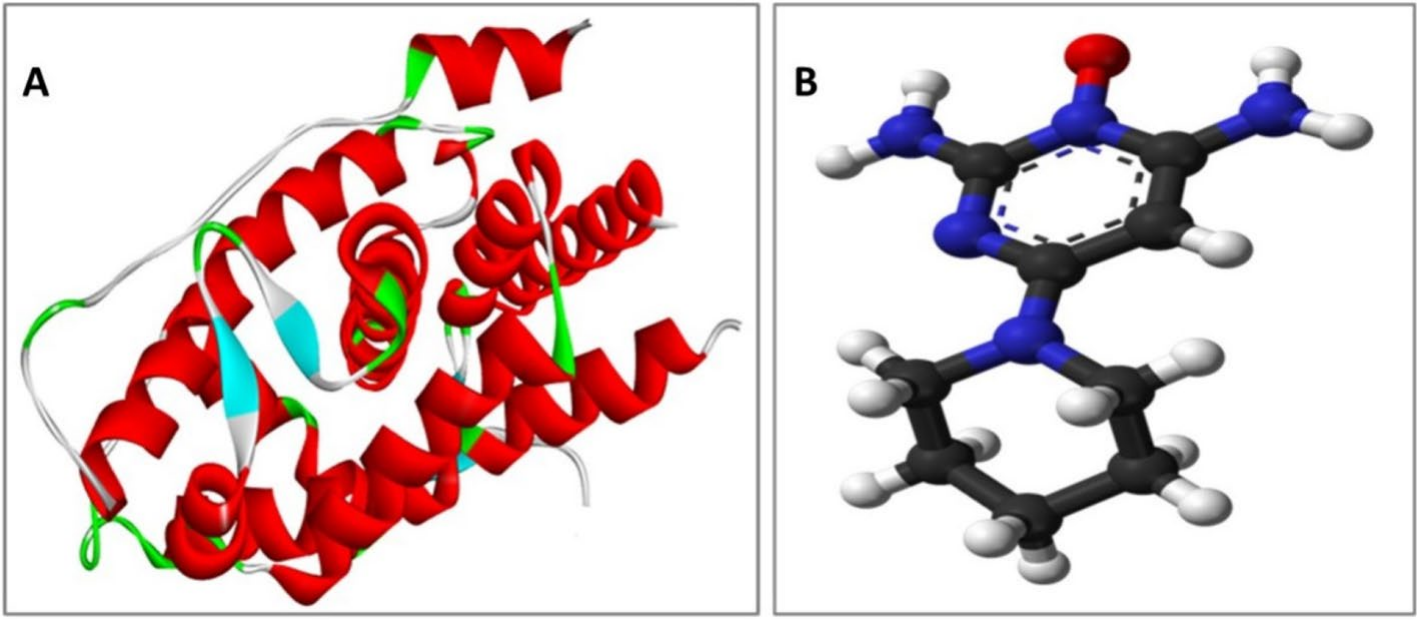
(**A**) Androgen receptor (4K7A), (**B**) Minoxidil which has been separated from its receptor.

The crystal structure of the androgen receptor in complex with minoxidil (PDB code:4K7A) was downloaded from http/www/pdbbeta.rscb.org with a resolution of 2.44 Ǻ. As DHT is a natural ligand that causes baldness and minoxidil is a drug used for the treatment of baldness, we used the minoxidil position in the crystal structure of the androgen receptor as the position for validation throughout the docking analysis. The receptor was 3D protonated, where hydrogen atoms were added at their standard geometry, the partial charges were computed, and the system was optimized. Deletion of co-crystallized water molecules was performed. The binding pocket had been defined and isolated (Fig. 2).

#### Validation of the molecular docking method

**Figure 3:**
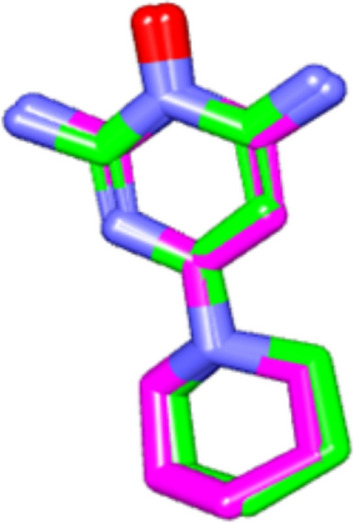
The alignment between the X-ray bioactive conformer of the minoxidil (colored in purple) and the redocked pose of the same compound (colored in green) at androgenic receptor binding site (PDB ID:4k7a).

To ensure the accuracy of the docking protocol, validation of the molecular docking method in this study was performed by re-docking minoxidil in the active site of the target protein using AutodockVina and visualized by Discovery Studio Visualizer. This was followed by the alignment of the X-ray bioactive conformer of the minoxidil with the best fitted pose achieved from docking. The alignment showed good coincidence between them, indicating the ability of the used docking protocol to retrieve valid docking poses. The method was deemed successful if the RMSD value returned was ≤ 2 Å ^15^ (Fig. 3).

#### Preparation of ligand structure

Test ligand structures of LSLE compounds, minoxidil and the reference ligand finasteride were prepared using AutoDock Vina. The ligand setup was employed to add gasteriger charges, find aromatic/aliphatic carbons, detect rotatable bond, and set torsion angle. Finally, the compounds were saved as PDBQT format.

#### Running docking protocol

The docking analyses of the identified molecules with androgenic receptor (4K7A) were carried out using AutoDock Vina. The program was run using a searching grid. The grid was typically over the androgenic receptor and had a size of 20 Å × 20 Å × 20 Å. The other parameters were set as default. The center of the active site was determined and presented as a 3D grid, with X = − 24, Y = 1 and Z = − 3, respectively. Compounds were docked into the crystal structure of 4K7A, and the highest scoring pose was selected for each of the compounds. The most stable conformation of each compound for binding to the protein active site was taken as the optimal docking posture.

## Results

### LC-MS/MS of LSLE

In folk medicine, heat is usually implemented in the extraction of *L. sativum* seeds. Accordingly, there is a higher probability for the loss of active constituents, particularly glucosinolates^16^. These are ubiquitous metabolites of the Brassicaceae plants known for their hair growth promoting potential via the degradation of dihydrotestosterone as in case of sulforaphane^17^. Therefore, the use of heat was avoided, and seeds were soaked in cold water, then lyophilized to form LSLE.

**Figure 4:**
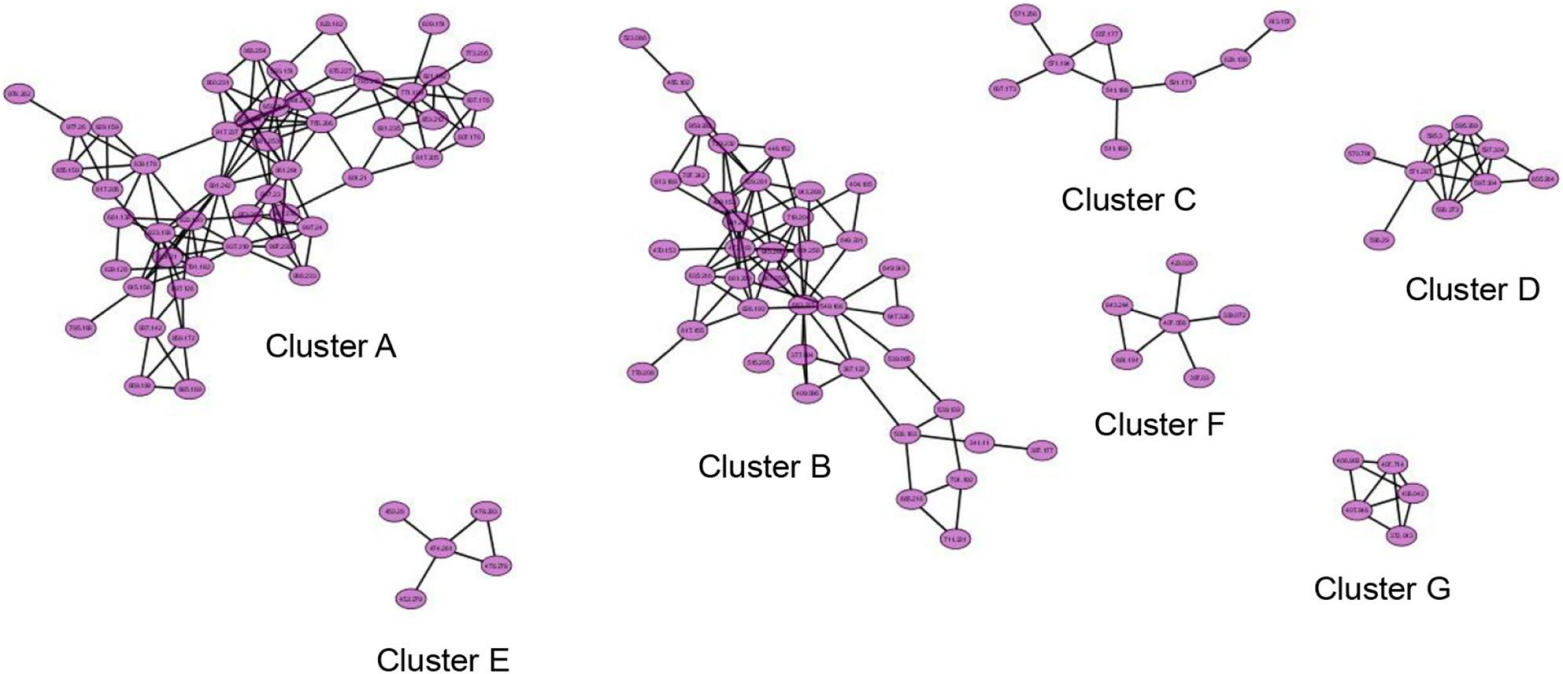
The enlarged significantly dereplicated clusters of negative molecular network created using MS/MS data (negative mode).

**Figure 5:**
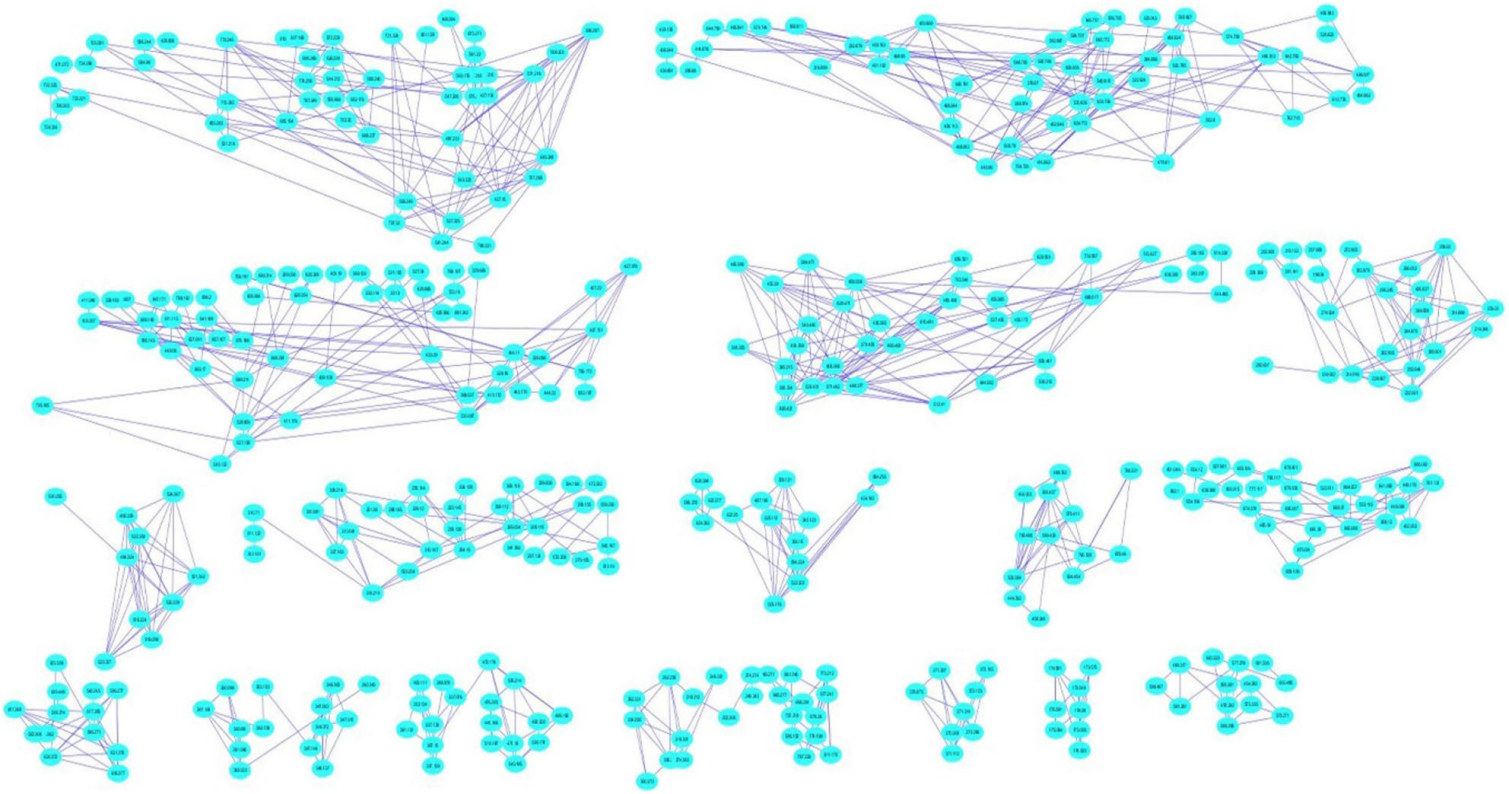
The clusters of positive molecular network created using MS/MS data (positive mode).

LC–MS/MS is an effective tool for the identification and dereplication of plant secondary metabolites^9,18^. Since plant extracts are constituting a complex matrix of diverse secondary metabolites with different chemical scaffolds that render their ionization behaviour different according to structure, we used both positive and negative ionization modes. Several glucosinolates has been reported and identified by LC–MS from plant extracts^19,20^. Data analysis resulted in the identification of 17 main compounds. Three glucosinolates, namely, glucobrassicanapin (**14**), glucotropeolin (**5**), sinigrin (**3**), seven flavonoids, namely, acacetin-7-*O*-rutinoside (**16**), catechin (**10**), luteolin-di-*O*-hexoside (**13**), quercetin 3-(6-*O*-acetyl-hexoside) (**8**), quercetin 3-*O*-deoxyhexose-hexose-7-O-deoxyhexose (**11**), quercetin 3-rutinoside-7-hexoside (**9**), syringetin-3-*O*-hexoside (**15**), a phenolic acid, sinapic acid (**2**), together with its glucoside (**12**), semilepidinosides A and B (**4** and **7**), the alkaloid lepidiene E (**6**), the coumarin esculin (**1**), and linolenic acid (**17**) were detected (Table 1 and Fig. S1). LC–MS/MS analysis followed by molecular networking of LSLE helped leveraging the metabolites of *L. sativum* seeds. Molecular networks (MNs) for the negative and positive ionization modes were created via GNPS platform (Global Natural Products Social Molecular Networking), where MN displayed the chemical space acquired from MS–MS fragmentation patterns similarity to enable the correlation of probably similar metabolites. This correlation would accelerate sorting and dereplication of constituents of the metabolome^21^. The negative MN resulted in 114 nodes grouped as 7 clusters (Fig. 4). They were dereplicated as a set of Clusters were cluster **A** (flavonoids), cluster **B** (sugars), cluster **C** (phenolic acid glycosides), cluster **D** (myoinositol derivatives), cluster **E** (fatty acid), cluster **F** (phenolic acid esters) and cluster **G** (glucosinolates). On the other hand, positive MN revealed similar results (Fig. 5). glucosinolates were the major compounds identified in the extract, followed by flavonoid glycosides, phenolic acids derivatives and alkaloids.

**Table 1:** Phytochemical Profile of LSLE by LC–MS/MS Analysis (Negative and Positive Ionization Modes).

	Rt (min)	Precursor m/z	Name	Formula	Adduct ion	MS/MS spectrum	Refs.
1	1.19	339.1956	Esculin	C_15_H_16_O_9_	[M-H]^-^	339.07, 179.07, 149.02, 121.02, 71.01	^22^
2	1.19	223.0612	Sinapic acid	C_11_H_12_O_5_	[M-H]^-^	223.06, 193.01,149.92, 121.03	^23^
3	1.23	358.0272	Sinigrin	C_10_H_17_NO_9_S_2_	[M-H]^-^	358.02, 313.12, 259.02, 96.95	^24^
4	1.32	337.1394	Semilepidinoside A	C_16_H_20_N_2_O_6_	[M + H]^+^	337.13, 217.09, 175.08, 81.04	^25^
5	1.49	408.0429	Glucotropeolin	C_14_H_19_NO_9_S_2_	[M-H]^-^	408.04, 212.00, 195.03, 166.03, 96.95, 74.99	^25^
6	1.91	347.1502	Lepidine E	C_20_H_18_N_4_O_2_	[M + H]^+^	347.15, 279.11, 173.07, 157.07, 81.04	^25^
7	2.69	365.1354	Semilepidinoside B	C_17_H_22_N_2_O_7_	[M-H]^-^	365.05, 212.93, 203.08, 187.05, 161.04,123.04	^26^
8	3.55	507.1133	Quercetin 3-(6-O-acetyl-hexoside)	C_23_H_22_O_13_	[M + H]^+^	507.21, 205.09	^27^
9	4.63	771.1989	Quercetin 3-rutinoside-7-hexoside	C_33_H_40_O_21_	[M-H]^-^	771.19, 625.13, 447.09, 299.01	^28^
10	4.68	289.0717	Catechin	C_15_H_14_O_6_	[M-H]^-^	289.07, 245.08, 123.04, 109.02	^29^
11	4.93	757.2185	Quercetin 3-O-deoxyhexose- hexose-7-O- deoxyhexose	C_33_H_40_O_20_	[M + H]^+^	757.21, 595.16, 433.11, 287.05	^30^
12	5.11	385.1140	Sinapoyl hexoside	C_17_H_22_O_10_	[M-H]^-^	385.11, 223.06, 205.05, 175.00	^23^
13	5.57	611.1574	Luteolin-3', 7-di-O-hexoside	C_27_H_30_O_16_	[M + H]^+^	611.15, 449.10, 303.05	^31^
14	5.59	410.0555	Glucobrassicanapin	C_12_H_21_NO_9_S_2_	[M + Na]^+^	410.05, 378.14, 276.10, 168.04, 91.05	^32^
15	6.61	507.1884	Syringetin-3-O-hexoside	C_23_H_24_O_13_	[M-H]^-^	507.12, 339.18, 102.95	^33^
16	9.34	591.1719	Acacetin-7-O-rutinoside	C_28_H_32_O_14_	[M-H]^-^	591.16, 325.09, 265.07, 205.05	^25^
17	19.96	277.2179	Linolenic acid	C_18_H_30_O_2_	[M-H]^-^	277.21, 259.20, 233.22	^25^

**Figure 6:**
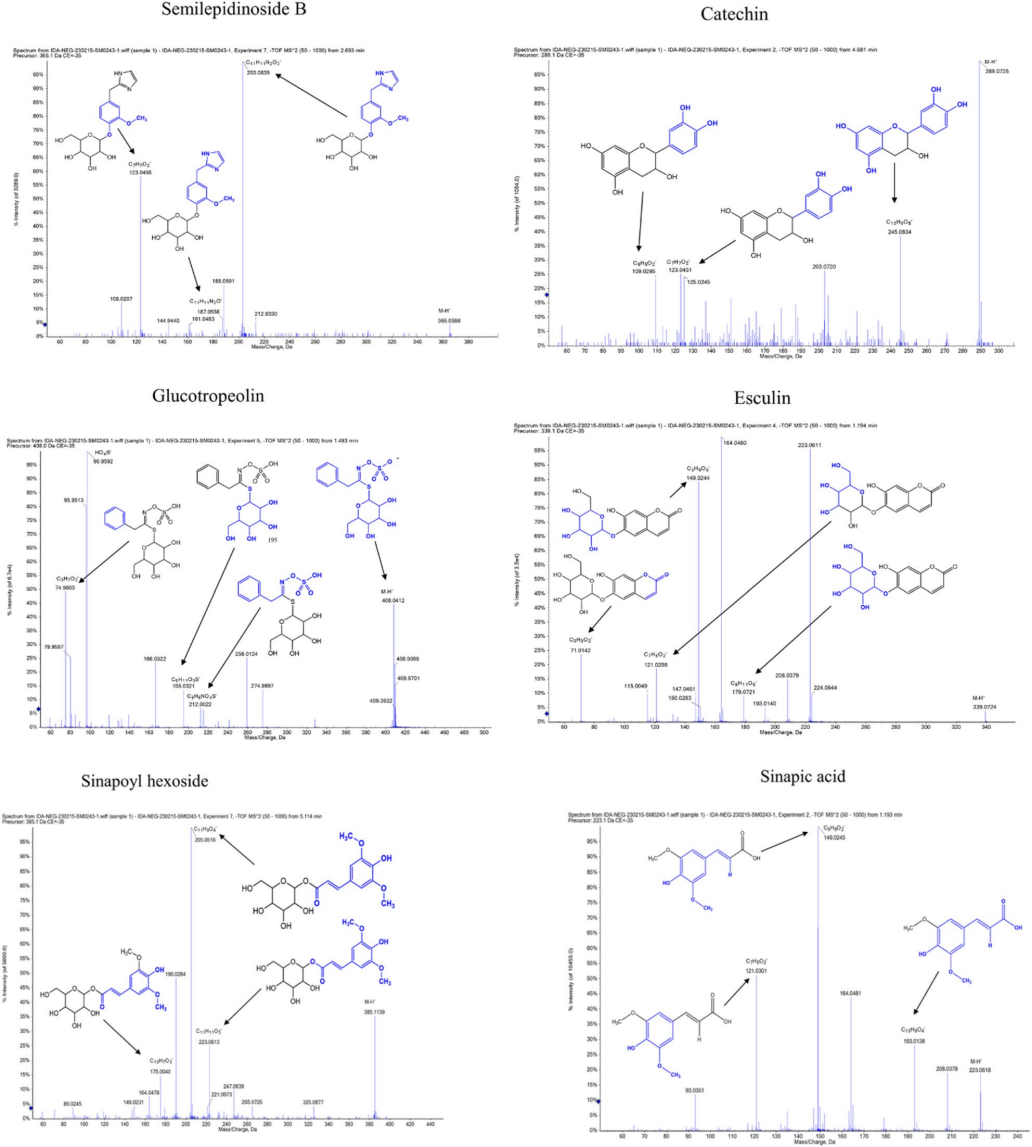
Major compounds identified in negative ion mode showing the MS/MS (MS^2^ fragmentation) of each compound, blue colored partial structures represent the lost fragments for each peak.

**Figure 7:**
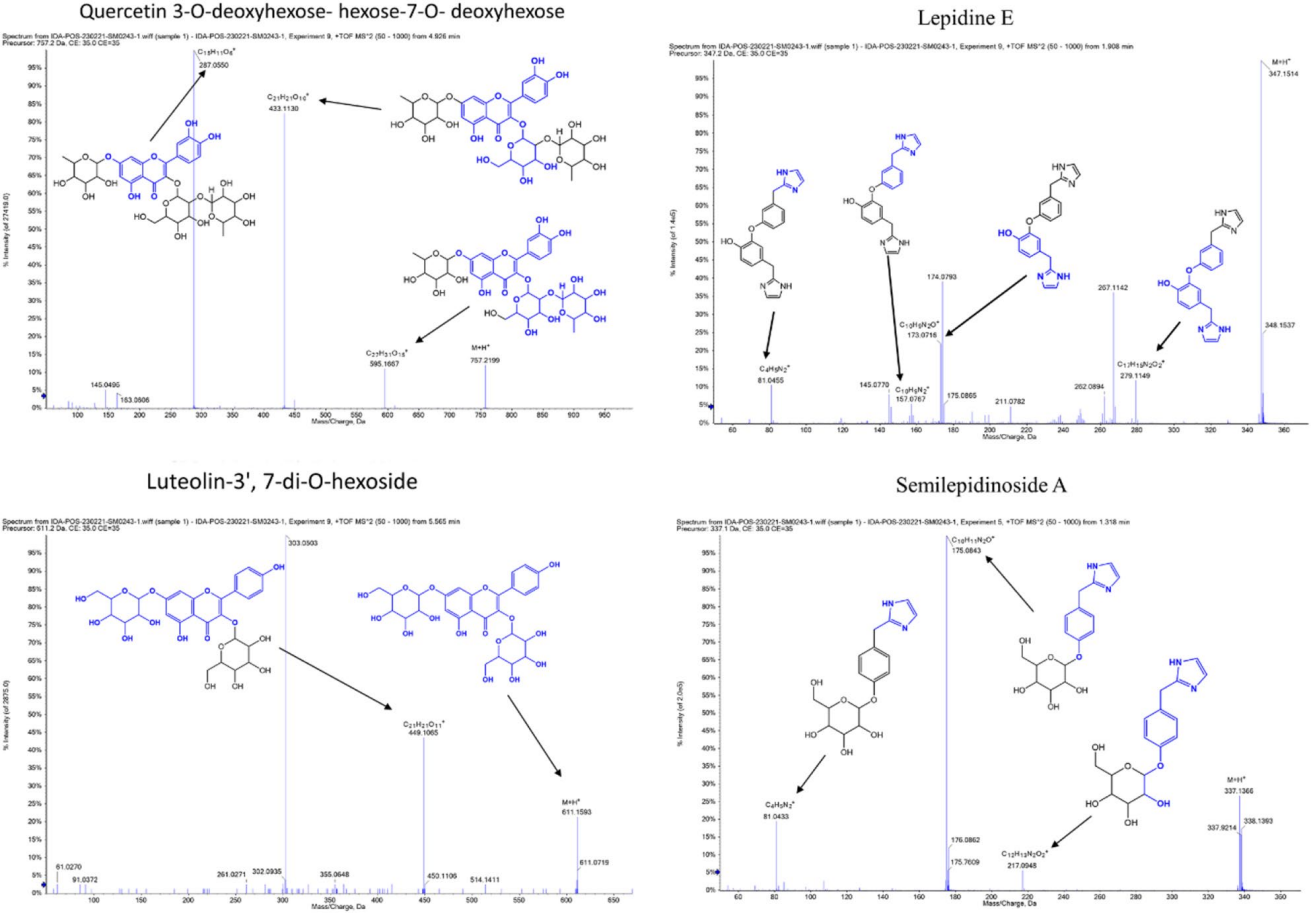
Major compounds identified in positive ion mode showing the MS/MS (MS^2^ fragmentation) of each compound, blue colored partial structures represent the lost fragments for each peak.

The major compounds identified from LSLE and their corresponding fragmentation patterns are illustrated in Fig. 6 for negative ion mode and Fig. 7 for positive ion mode.

### Biochemical parameters

#### Effect of LSLE on cholesterol levels in rats with induced alopecia; treatment and withdrawal

**Figure 8:**
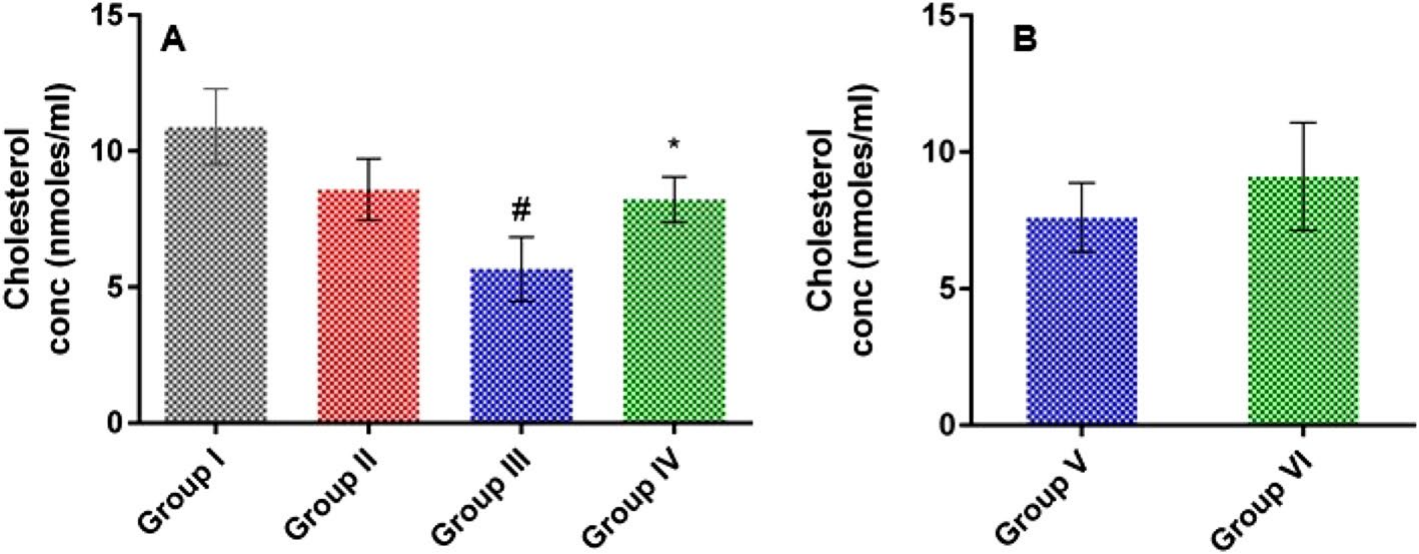
Effect of LSLE and minoxidil on serum cholesterol levels in Sustanon induced alopecia in rats. (**A**) Effect of treatment. Group I had the highest mean of serum cholesterol with a concentration of 10.9 ± 1.40 nmol/ml. The group receiving Sustanon had a decreased level of 8.9 ± 1.13 nmol/ml. Administration of minoxidil decreased serum cholesterol further to 5.66 ± 1.18 nmol/ml. LSLE had similar cholesterol mean to group II with a concentration of 8.21 ± 0.83 nmol/ml. (**B**) Effect of stopping the treatment. Both groups, V and VI had their cholesterol levels elevated with no significant difference between them. Results represent mean ± SD (n = 7). Statistical analysis was done using one-way ANOVA followed by Tukey’s post hoc test. # refers to a significant difference from the untreated group (*p* < 0.05). * Refers to a significant difference from the minoxidil treatment group (*p* < 0.05).

The administration of Sustanon showed an approximately 20% decrease in the level of cholesterol compared to the control group. Treatment with LSLE did not affect the cholesterol level while minoxidil significantly decreased the cholesterol level. After stopping the treatment, the level of cholesterol increased in both groups. The level of cholesterol was insignificantly higher in the LSLE than in the minoxidil group (Fig. 8).

#### Effect of LSLE on 5AR levels in rats with induced alopecia; treatment and withdrawal

**Figure 9:**
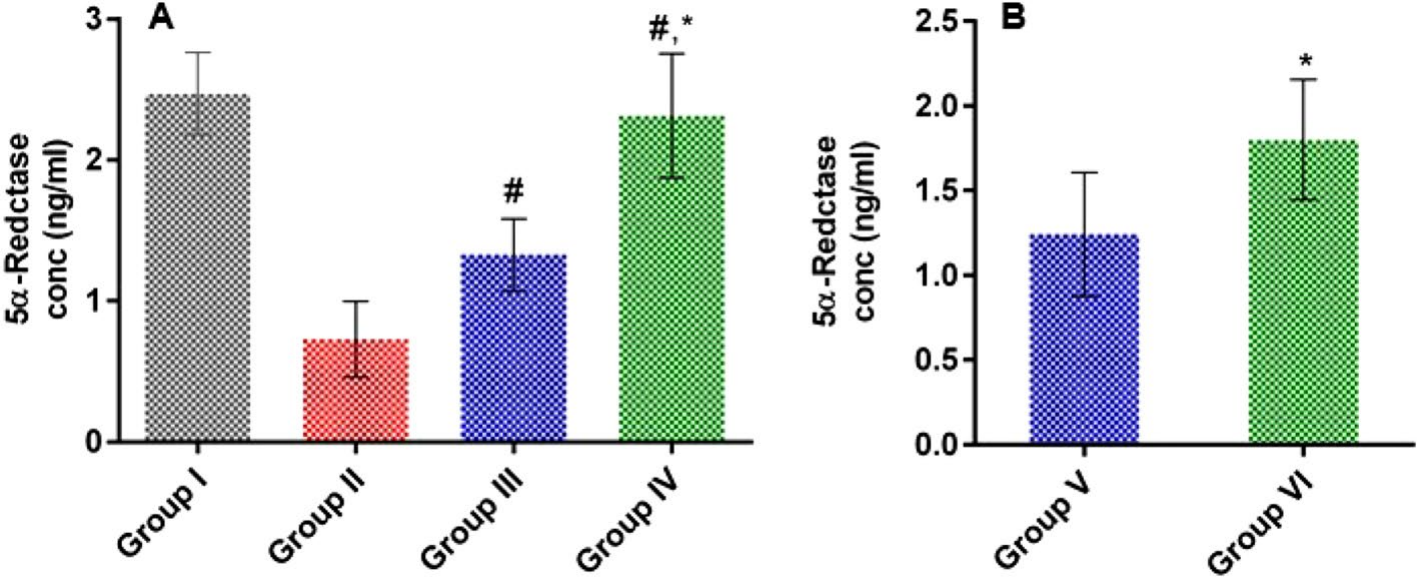
Effect of LSLE and minoxidil on tissue levels of 5AR in Sustanon induced alopecia in rats. (**A**) Effect of treatment. Group I had the highest level of tissue 5AR with a concentration of 2.47 ± 0.29 ng/ml. Administration of Sustanon resulted in decreasing the 5AR level to 0.73 ± 0.27 ng/ml. Treatment with minoxidil and LSLE increased the 5AR levels to 1.33 ± 0.26 ng/ml and 2.31 ± 0.44 ng/ml respectively. Results represent mean ± SD (n = 7). (**B**) Effect of stopping the treatment. The 5AR levels decreased slightly to 1.24 ± 0.364 ng/ml in group V compared to 1.8 ± 0.356 ng/ml in group VI. Statistical analysis was done using one-way ANOVA followed by Tukey’s post hoc test. # refers to a significant difference from the untreated group (*p* < 0.05). * Refers to a significant difference from the minoxidil treatment group (*p* < 0.05).

The administration of Sustanon significantly decreased tissue 5AR compared to the control group. After 21 days, topical minoxidil increased serum 5AR by more than 1.8 folds while LSLE increased it by 3.16 folds compared to the Sustanon group. In groups V and VI where the treatment was stopped, the levels of 5AR fell more in the LSLE compared to the minoxidil but remained significantly higher (Fig. 9).

#### Effect of LSLE on CTGF in rats with induced alopecia; treatment and withdrawal

**Figure 10:**
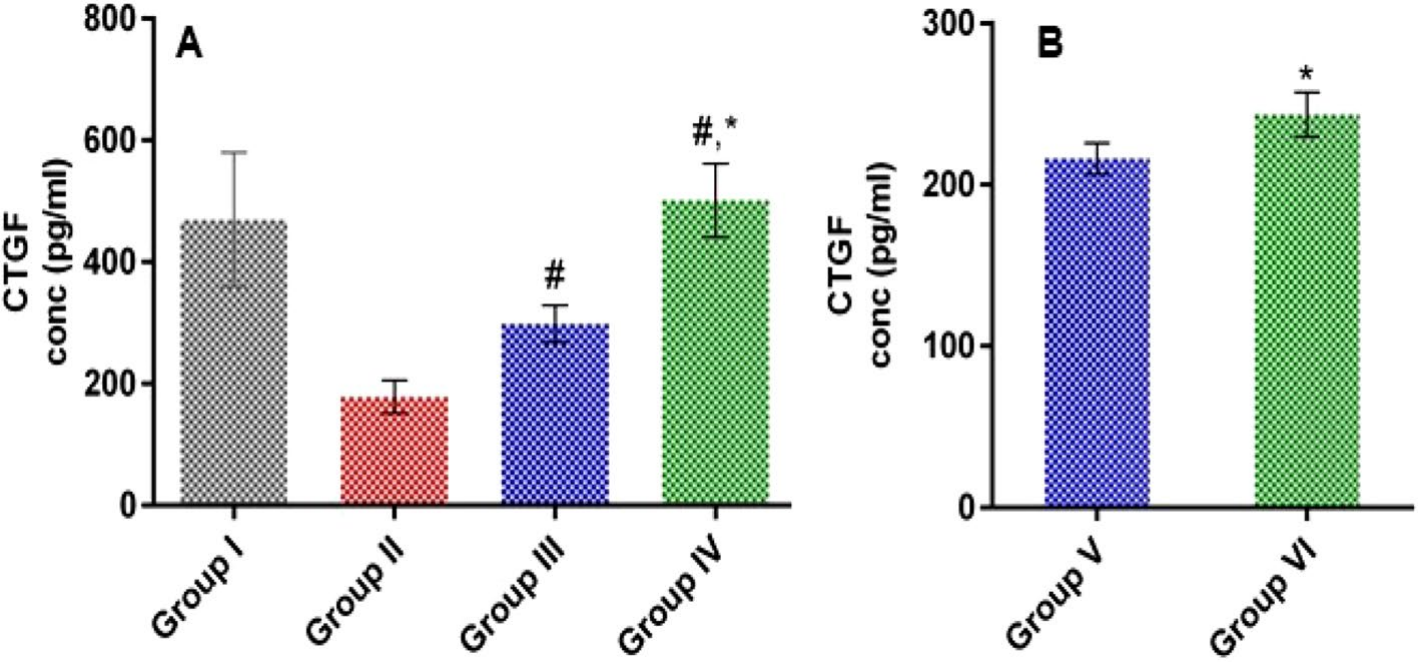
Fig. 10: Effect of LSLE and minoxidil on tissue levels of CTGF in Sustanon induced alopecia in rats. (**A**) Effect of treatment. Group I had the highest level of tissue CTGF with a concentration of 469.94 ± 110.63 pg/ml. Administration of Sustanon resulted in decreasing the CTGF level to 178.21 ± 27.13 pg/ml. Treatment with minoxidil and LSLE increased the CTGF levels to 298.63 ± 30.37 pg/ml and 501.47 ± 60.24 pg/ml respectively. (**B**) Effect of stopping the treatment. The level of CTGF started to decrease back to 216.1 ± 9.77 pg/ml and 243.4 ± 13.79 pg/ml with groups V and VI respectively. Results represent mean ± SD (n = 7). Statistical analysis was done using one-way ANOVA followed by Tukey’s post hoc test. # Refers to a significant difference from the untreated group (*p* < 0.05). * Refers to a significant difference from the minoxidil treatment group (*p* < 0.05).

Administration of Sustanon for 21 days significantly reduced the CTGF levels in group II compared to the control group by 2.64 folds. The topical coadministration of minoxidil with the S.C of Sustanon increased CTGF by 1.68 folds compared to group II. Treatment with LSLE increased the CTGF to exceed that of the control group to reach 501.47 ± 60.24 pg/ml. After stopping the treatment, the level of CTGF decreased in both groups V and VI but the decrease was 48.5% in the LSLE group compared to only 28% in the minoxidil group. The concentration remained relatively higher in the LSLE group (Fig. 10).

#### Effect of LSLE on FGF in rats with induced alopecia; treatment and withdrawal

**Figure 11:**
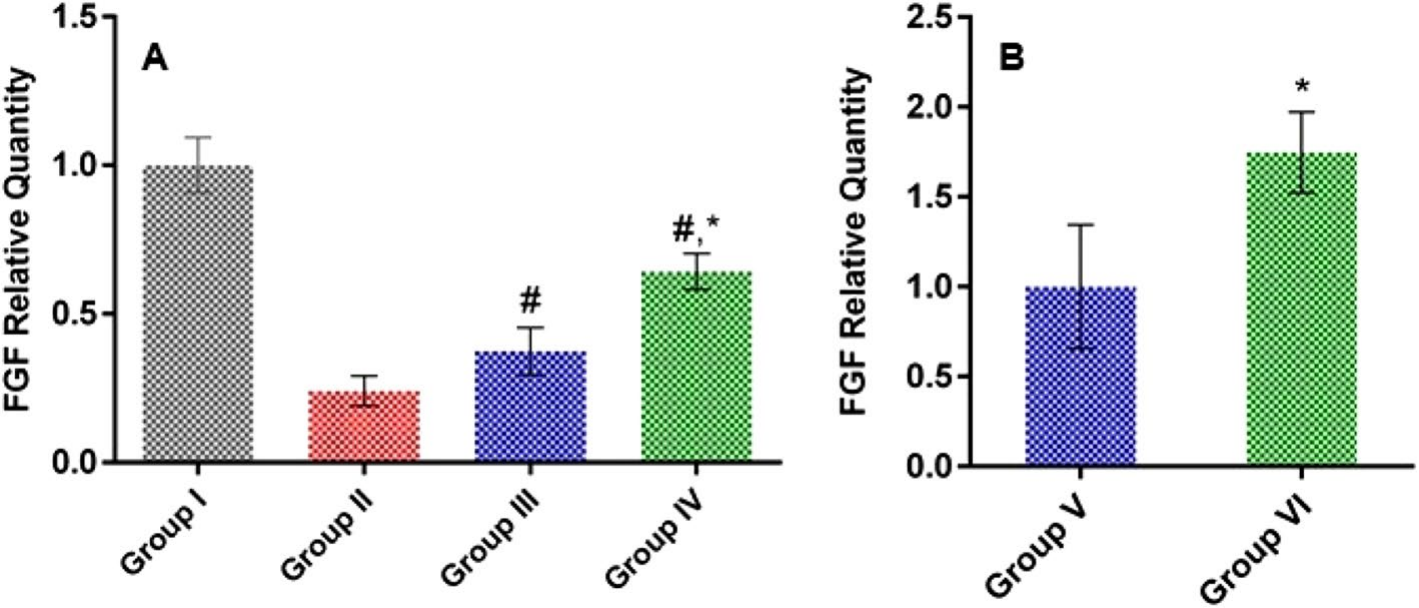
Effect of LSLE and minoxidil on tissue levels of FGF in Sustanon induced alopecia in rats. (**A**) Effect of treatment. Administration of Sustanon resulted in decreasing the FGF levels to 0.24 ± 0.05 of the control group. Treatment with minoxidil and LSLE increased the FGF levels to 0.37 ± 0.08 and 0.64 ± 0.06 of the control group respectively. (**B**) Effect of stopping the treatment. The results were normalized relative to the minoxidil group. The concentration was relatively higher in the LSLE group than in the minoxidil group. Results represent mean ± SD (n = 7). Statistical analysis was done using one-way ANOVA followed by Tukey’s post hoc test. # Refers to a significant difference from the untreated group (*p* < 0.05). * Refers to a significant difference from the minoxidil treatment group (*p* < 0.05).

FGF levels were measured by relative quantification against the housekeeping gene (β-actin) per tissue sample and relative to the control group. The group of induced alopecia manifested a significant reduction in the FGF levels by 76% compared to the control group. Nevertheless, both treated groups showed higher FGF levels. Group III was 13% higher while group IV was 40% higher compared to group II. On discontinuing the treatment, the levels of FGF were normalized to the minoxidil group (group V). The level of FGF was 75% higher in group VI compared to group V (Fig. 11).

#### Effect of LSLE on VEGF in rats with induced alopecia; treatment and withdrawal

**Figure 12:**
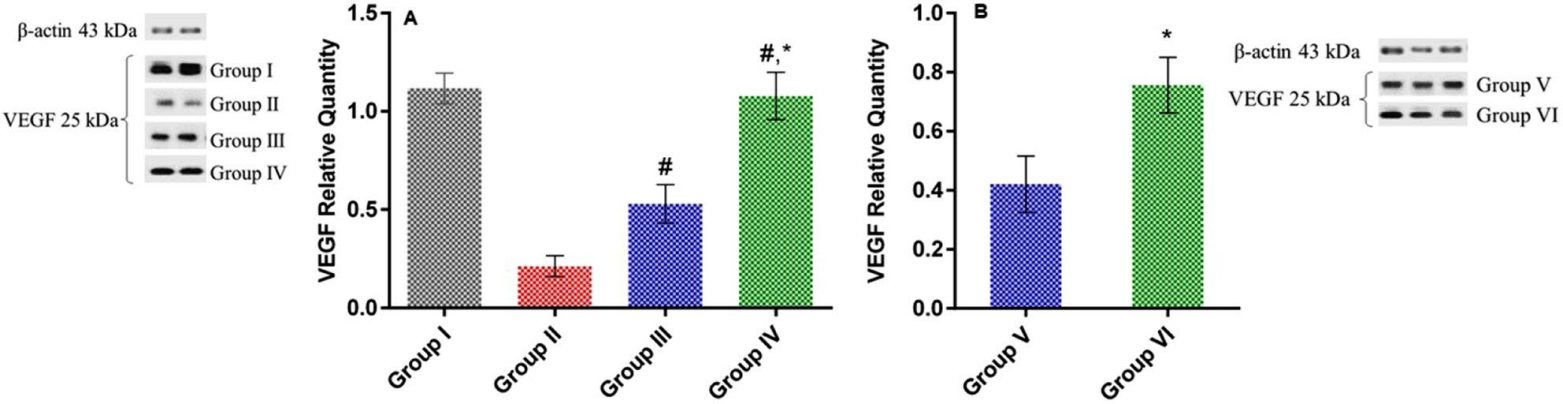
Effect of LSLE and minoxidil on tissue levels of VEGF in Sustanon induced alopecia in rats. (**A**) Effect of treatment. Group I had the highest level of tissue VEGF with a ratio of 1.12 ± 0.08 to β-actin. Administration of Sustanon resulted in decreasing the VEGF level to 0.21 ± 0.05. Treatment with minoxidil and LSLE increased the VEGF levels to 0.53 ± 0.1 and 1.08 ± 0.12 respectively. (**B**) Effect of stopping the treatment. The level of VEGF fell back to 0.42 ± 0.1 in group V and 0.76 ± 0.09. The concentration was relatively higher in the LSLE group than in the minoxidil group. Results represent mean ± SD (n = 7). Statistical analysis was done using one-way ANOVA followed by Tukey’s post hoc test. # Refers to a significant difference from the untreated group (*p* < 0.05). * Refers to a significant difference from the minoxidil treatment group (*p* < 0.05).

The administration of Sustanon decreased the VEGF expression levels by 5 folds compared to the control group. On treating the rats, the VEGF levels increased significantly by 2.5 folds in the minoxidil group and by 5 folds in the LSLE group compared to the untreated group. After stopping the treatment, the level of VEGF decreased in both groups, V and VI. The level in the minoxidil group decreased by 20% and in the LSLE by 25% (Fig. 12).

### Hair growth and histopathology

#### Hair growth

**Figure 13:**
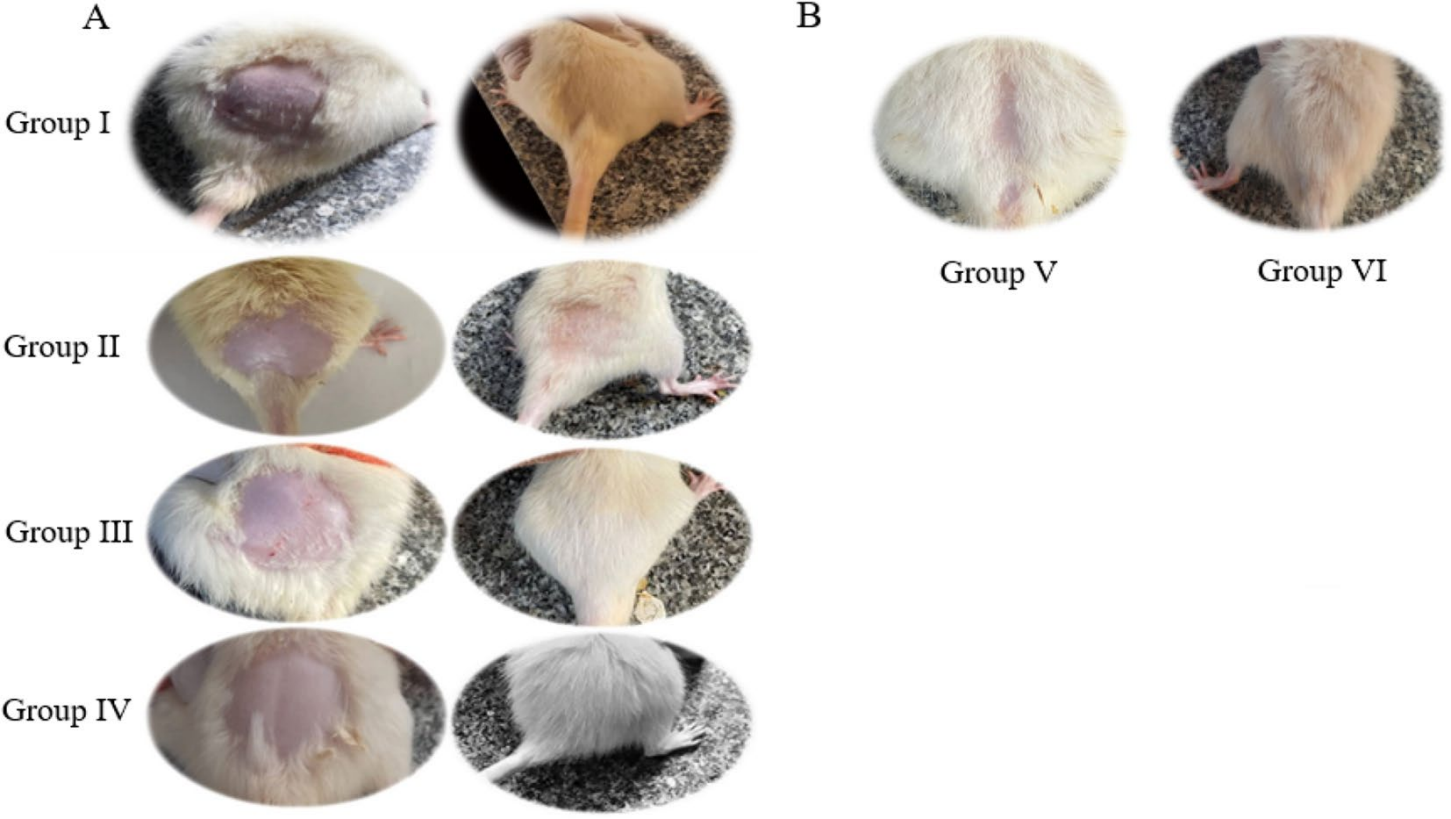
Effect of LSLE and minoxidil on hair growth rates in Sustanon induced alopecia in rats. (**A**) Effect of treatment. Treatment with minoxidil and LSLE increased the rate of hair growth compared to control group. LSLE showed the highest hair growth rate compared to minoxidil group. LSLE was considered effective hair growth stimulant than minoxidil. (**B**) Effect of stopping the treatment. LSLE group showed better results than minoxidil group after withdrawal. It can reduce hair loss and maintain hair growth.

Hair growth rates were observed daily and by comparing the hair growth among the groups. The LSLE showed the highest hair growth rate (Fig. 13).

#### Histopathological assessment of hair follicles

**Figure 14:**
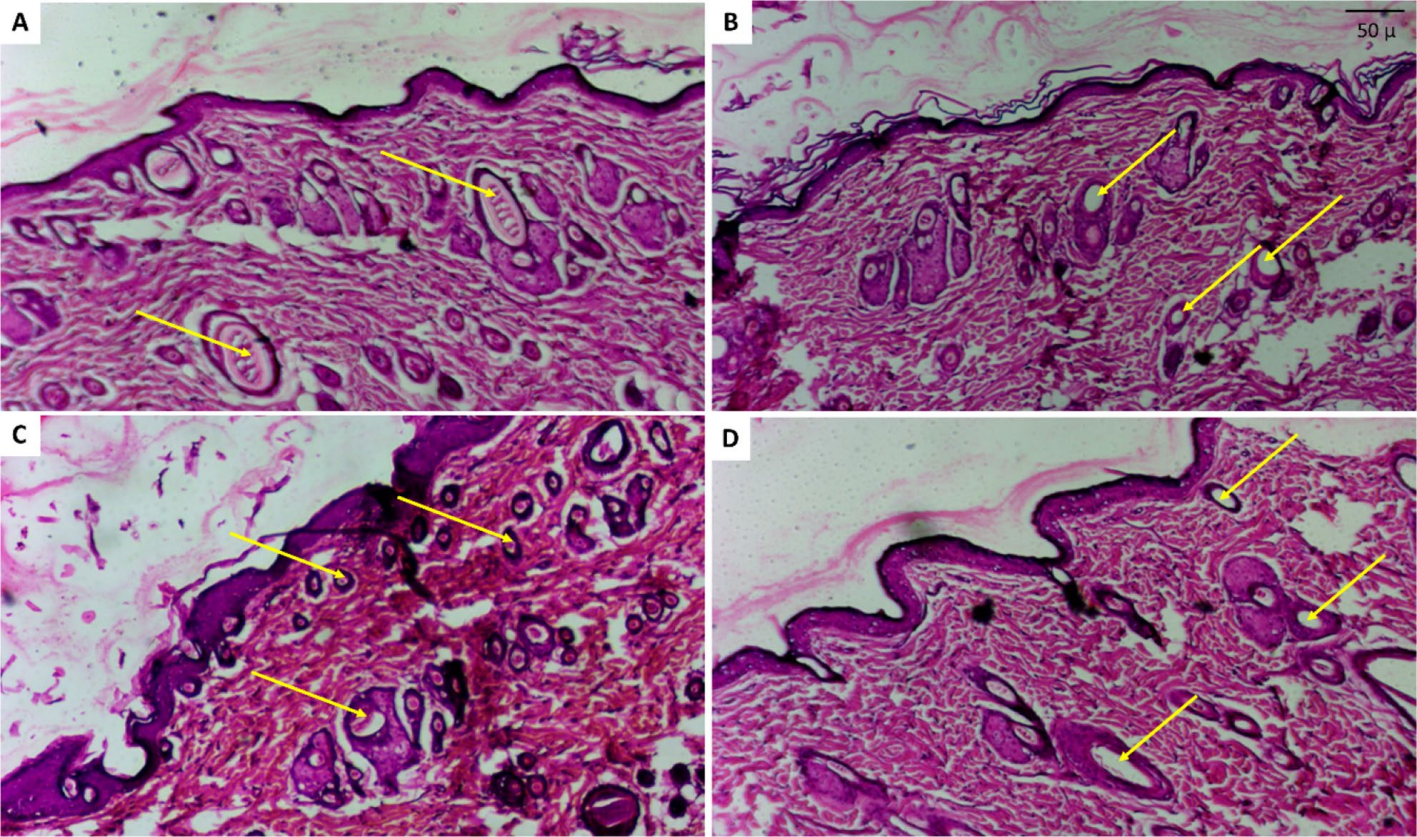
Photomicrography with H&E stained transverse sections of rat’s skin: (**A**) Normal control group showing abundant hair follicles in normal growth phase “yellow arrows”, (**B**) Resting dorminant follicles (i.e. damaged hair follicles no longer able to grow to a developed hair shaft) in control positive group “yellow arrows” (**C**) Lepidium tested group with high number of active growing follicles resembling normal group “yellow arrows” (**D**) Minoxidil tested group visualizing multiple empty follicles than found in Lepidium group but less than control positive group “yellow arrows”.

**Figure 15:**
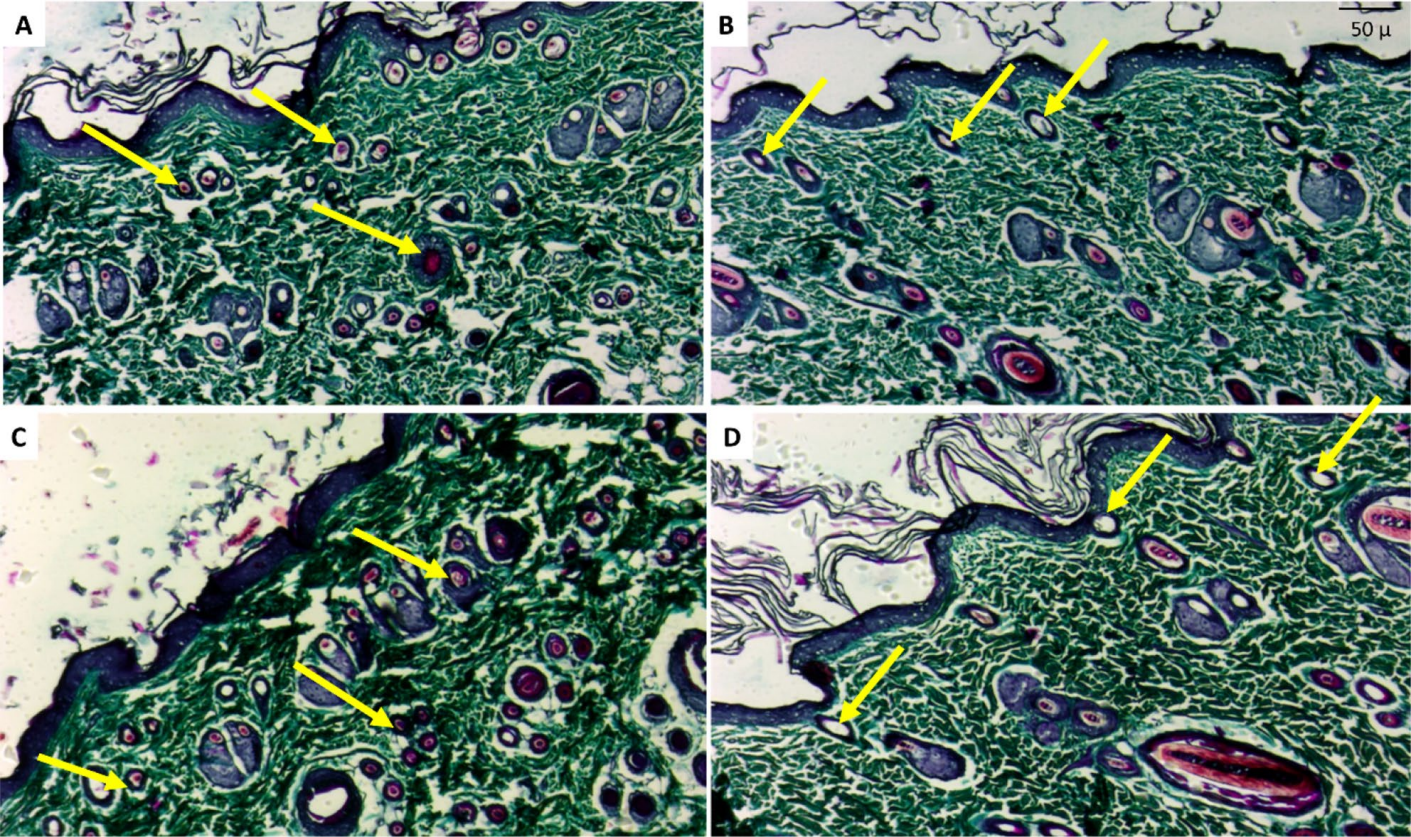
Photomicrography with Masson trichrome stain transverse sections of rat’s skin: (**A**) Normal control group showing the active hair follicles “yellow arrows” with deeply stained collagen around in blue green color, (**B**) Resting dorminant follicles in control positive group that are empty with no further hair follicles “yellow arrows” (**C**) LSLE tested group with higher presence of active growing follicles resembling normal tested groups “yellow arrows” (**D**) Minoxidil tested group visualizing multiple empty follicles exceeding that found in the LSLE group but much less than control positive group “yellow arrows”.

Transverse sections are a very promising and reliable approach for confirming the diagnosis because all hair follicles can be clearly visualized, so all hair follicles were counted on transverse sections for greater accuracy. The total number of hair follicles may be unaffected, but the effect was observed as progressive miniaturization in the already present hair in the control positive group, as well as a difference in the size of hair follicles and expanded vellus hairs (growing hair- thin yellow arrows) in the tested groups. The total number of hair follicles counted along 10 examined fields in each group was statistically calculated (Figs. 14 and 15). The findings were confirmed on Masson trichrome stained sections, which allowed for a better view of the growing hair follicles.

**Figure 16:**
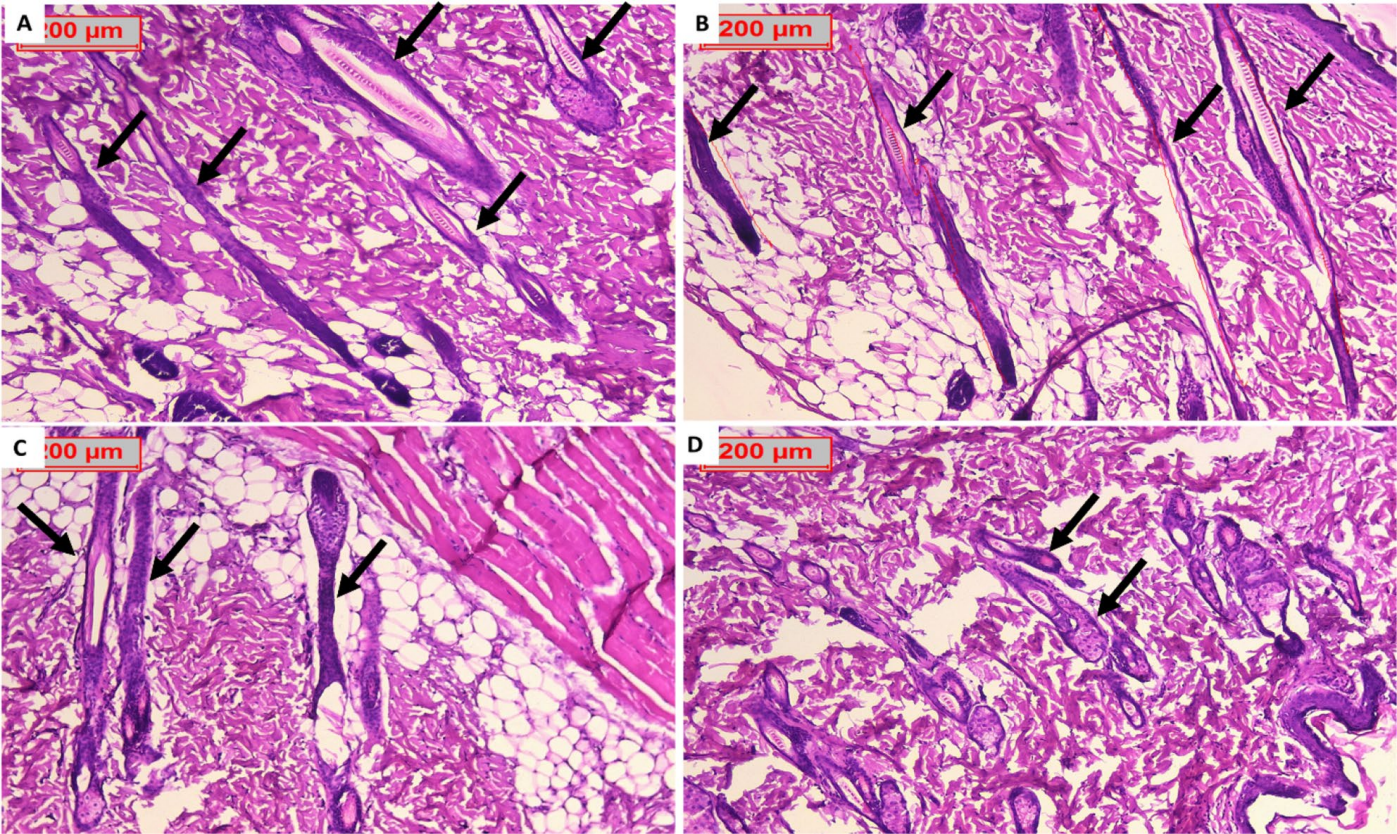
H&E stain. (**A**) Represent photomicrography of LSLSE, transverse section, counting the growing hair follicles. (**B**) Represent photomicrography of LSLSE, longitudinal section, counting the length of the follicles. (**C**) Represent photomicrography of minoxidil, transverse section, visualizing the growing hair follicles. (**D**) Represent photomicrography of minoxidil, longitudinal section, visualizing the length of the follicles. LSLE showed higher number of growing follicles as well as linger hair shafts of the already established fully formed hairs. All were photographed by magnification 100x, scale bar 200 µm.

**Figure 17:**
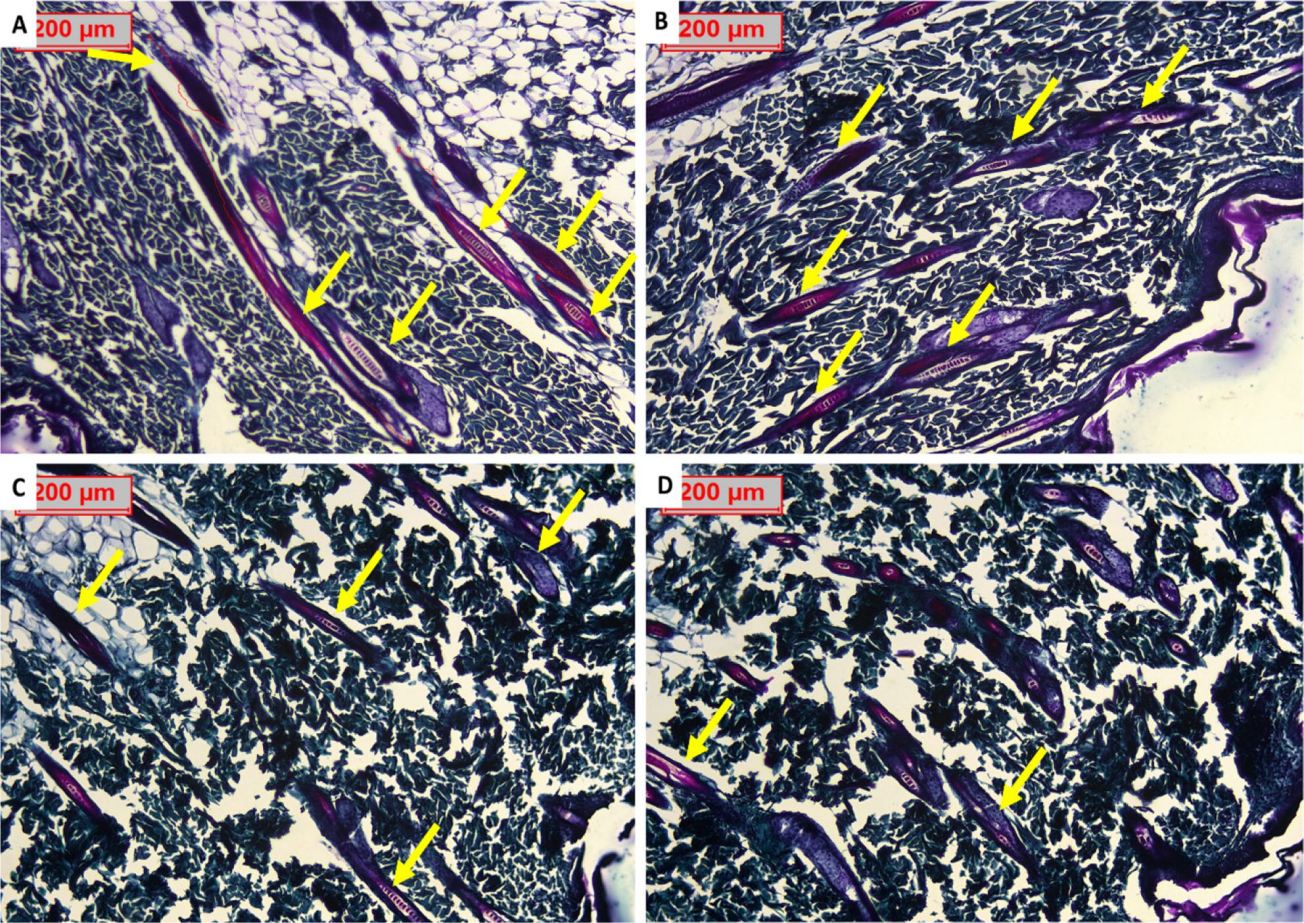
(**A**) Represent photomicrography of LSLE, transverse section, visualizing the growing hair follicles. (**B**) Represent photomicrography of LSLE, longitudinal section, visualizing the length of the follicles. (**C**) Represent photomicrography of minoxidil transverse section, visualizing the growing hair follicles. (**D**) Represent photomicrography of minoxidil longitudinal section, visualizing the length of the follicles. LSLE showed a higher number of growing follicles as well as linear hair shafts. Masson trichrome stain provided better observation and judging of the collagen, which is stained blue green, being deeper in LSLE treated groups than minoxidil treated groups. All were photographed by magnification 100x, scale bar 200 μm.

On continuation of the study for the further effect of both drugs, H&E-stained sections of treated groups revealed excess hair follicular growth as well as long hair shafts with a privilege of LSLE over minoxidil treated groups. Representative Masson trichrome microscopic images confirmed the ordinary H&E results with better visualization of dermal papillae, hair shafts and bulbs (Figs. 16 and 17).

### Docking study

**Figure 18:**
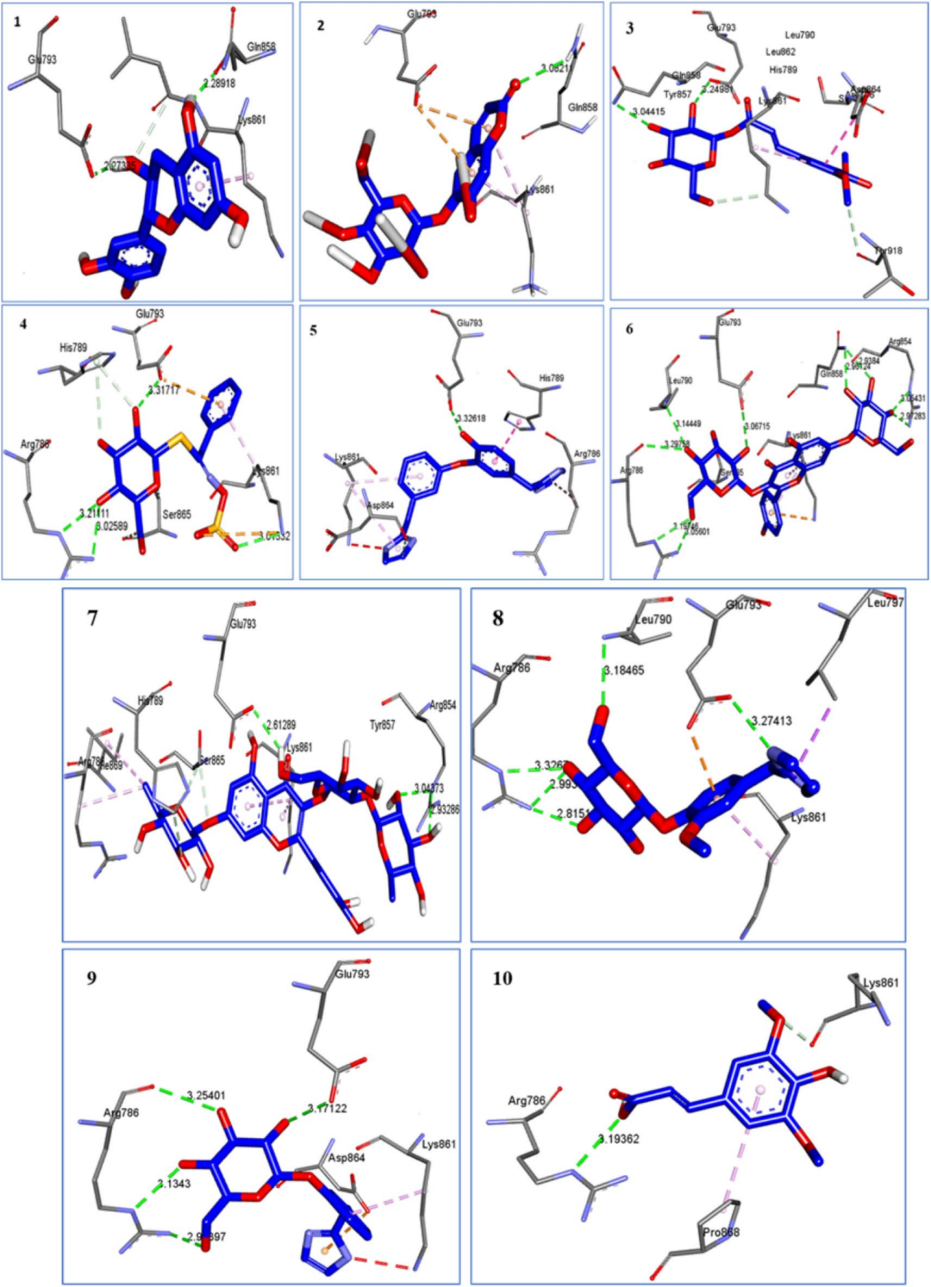
Visualization of interactions between the androgen receptor (4K7A) and ten major compounds identified showed comparable or better binding energy and binding modes than that of minoxidil (6.9 kcal/mol) as well as that of the gold standard therapy compound, finasteride.

Docking simulation of the major ten compounds identified showed that they fit into the receptor active site almost at the same position of minoxidil with comparable docking scores ranging (from − 4.1 to − 8.9 kcal/mol, in comparison with − 6.9 kcal/mol for minoxidil) (Fig. 18, Table 2).

**Table 2:** Summarizes docking scores and H-bond interactions of the target compounds and Minoxidil with amino acids of androgenic receptor.

	Compound	Binding energy	Hydrogen bond distance (A)	Hydrogen bonds	Nearest amino acid residues
	Minoxidil	− 6.9	2.21	GLU^793^	GLN^858^, SER^865^, LYS^861^
	Finasteride	− 6.3	2.02 and 2.32	ARG^854^	GLU^793^, TRY^857^, GLN^858^
1	Catechin	− 6.6	2.27 2.28	GLU^793^ GLN^858^	LYS^861^, LEU^862^
2	Esculin	− 6.9	3.08	GLN^858^	GLU^793^, LYS^861^
3	Sinapoyl hexoside	− 5.6	3.24 3.04	GLU^793^ GLN^858^	HIS^789^, TRY^857^, LEU^862^, LEU^790^, SER^865^, THR^918^
4	Glucotropeolin	− 5.7	3.31 3.01 3.02, 3.21	GLU^793^ LYS^861^ ARG^786^	HIS^789^, SER^865^
5	Lepidine E	− 4.1	3.32	GLU^793^	ARG^786^, HIS^789^, LYS^861^ , ASP^864^
6	Luteolin-3’,7-dihexoside	− 8.9	3.06 2.97, 3.05 2.93, 2.96 3.29, 3.19, 3.05 3.14	GLU^793^ ARG^854^ GLN^858^ ARG^786^ LEU^790^	LYS^861^, SER^865^
7	Quercetin 3-O-deoxyhexose- hexose-7-O- deoxyhexose	− 8.9	2.61 2.93, 3.04	GLU^793^ ARG^854^	TRY^857^, LYS^861^ SER^865^, HIS^789^
8	Semilepidinoside B	− 8.8	3.27 3.18 2.81, 2.99, 3.32	GLU^793^ LEU^790^ ARG^786^	LEU^797^, LYS^861^
9	Semilepidinoside A	− 8.4	3.17, 2.92, 3.13, 3.25	GLU^793^ ARG^786^	LYS^861^, ASP^864^
10	Sinapic acid	− 4.7	3.19	ARG^786^	LYS^861^, PRO^868^

Minoxidil and the androgen receptor form hydrogen bonds at GLU^793^, with LEU^862^, LYS^861^, and TYR^857^ being the four nearest amino acid residues. Different amino acid residues creating a hydrogen bond with the androgen receptor are factors that cause the binding energy for finasteride and identified compounds to be higher than minoxidil. Minoxidil forms hydrogen bonds with the androgen receptor's GLU^793^. Additional hydrophobic interactions with TRP^796^ and HIS^789^ influence ligand stability with the androgen receptor. Hydrophobic interactions, which repel liquid, are more likely to gather in protein globular form. Arginine is projected to play a crucial function in the androgen receptor ligand binding domain^34^.

Inspection of the top docking poses of the target compound (Luteolin-3’,7-di-*O*-hexoside) revealed that the O–H formed one hydrogen bond to the carboxylate of Glu^793^ (3.06 Å), while the other OH involved in hydrogen bond with the backbone NH of ARG^786^, LEU^790^, ARG^854^, GLN^858^ which essential in androgen receptor ligand binding.

## Discussion

Androgenic alopecia (AGA) is a difficult condition that affects both men and women. It is tolerated by some, but it lowers their self-esteem and has a negative psychological impact on them. This study was simply designed to investigate the therapeutic effect of LSLE on AGA and compare it to minoxidil 5%, which is available on the market and used to treat similar cases.

A modification in the extraction of the *L. sativum* seeds' active constituents was performed. The seeds were soaked in cold water and then lyophilized to form LSLE. The traditional use of heat treatment in folk medicine was avoided to prevent the loss of active constituents.

LC–MS/MS analysis is useful for the determination of secondary metabolites of plant extracts^19,20^. LC–MS/MS analysis of LSLE identified 17 compounds tentatively, glucosinolates, phenolic acids derivatives, alkaloids and flavonoids. The identified glucosinolates are glucobrassicanapin (**14**), glucotropeolin (**5**), and sinigrin (**3**). Flavonoid glycosides observed in LSLE were luteolin-di-*O*-hexoside (**13**), quercetin 3-(6-*O*-acetyl-hexoside) (**8**), quercetin 3-*O*-deoxyhexose-hexose-7-O-deoxyhexose (**11**), quercetin 3-rutinoside-7-hexoside (**9**), and syringetin-3-*O*-hexoside (**15**) the identified compounds are mono, di and triglucoside derivatives of quercetin and kaempferol, in addition to syringetin-3-*O*-hexoside. a phenolic acid, sinapic acid (**2**), together with its glucoside (**12**), semilepidinosides A and B (**4** and **7**), the alkaloid lepidiene E (**6**), the coumarin esculin (**1**), and linolenic acid (**17**) were also detected.

Glucosinolates are repeatedly reported to alleviate AGA conditions by enhancing the degradation of DHT^17^, inhibiting testosterone induced dermal papillae cells (DPCs) apoptosis^5^, stimulating DPCs proliferation and upregulating vascular endothelial growth factor (VEGF)^6^, or antiandrogenic activity^8^. Which postulates that the LSLE glucosinolates have a crucial role in promoting hair growth.

Meanwhile, flavonoids and phenolic acids are well known antioxidants that can alleviate oxidative stress. Oxidative stress in turn can cause many adverse effects on the skin and scalp that accelerate hair loss^35^.

On the other hand, docking study of ten representative constituents of LSLE against the androgen receptor (PDB code: 4K7A). revealed highest binding energy (ΔG) for the flavonoids luteolin-3’,7-dihexoside, quercetin 3-O-deoxyhexose- hexose-7-O- deoxyhexose, the alkaloidal glycosides semilepidinoside B and semilepidinoside A when compared to the standard drug minoxidil, which is mostly attributed to hydrogen bonding with amino acids that exist in vicinity of the ligand site, in addition to GLU^793^, which is the seldom hydrogen bonding residue with minoxidil.

For the aforementioned reasons regarding the diversity of metabolites in LSLE and its correlation to their antiandrogenic, antioxidant and predicted AR inhibitory activity, the use of the LSLE might be useful for the treatment of AGA without need for further fractionation or purification, due to the reciprocating potential of its diverse metabolome to aim multiple targets involved in the etiology of AGA.

Sustanon is made up of four different testosterone esters (testosterone propionate, phenylpropionate, isocaproate, and decanoate)^36^. These are simply short and intermediate esters with shorter absorption half-lives and higher clearance rates than longer chain esters^36^. As a result, Sustanon (a testosterone analogue) was used in our study to induce alopecia quickly.

The level of cholesterol was inversely proportional to the level of testosterone. This was reported by Zarei et al.^37^, and can be attributed to the conversion of the testosterone to 17-β-estradiol^38^. Although this remains controversial as other studies stated that testosterone may activate HMG-CoA reductase^39^, we were interested more in the fact that minoxidil reduced serum cholesterol. It was reported in a previous study that systemic minoxidil can affect cholesterol levels^40^ and so, it seems that there is partial absorption from its topical application. This was discussed before where around 1.7% of the applied minoxidil is systemically absorbed^41^. Which explains why the cholesterol levels were lower in groups III and V compared to groups IV and VI. This is advantageous to the LSLE as it showed no detected systemic effect.

5AR, which converts testosterone to DHT, is an important enzyme in determining the rate of androgenic alopecia. DHT has been shown to increase the expression of 5AR^42^, which explains the elevated level in group II. Minoxidil reduced 5AR concentrations, but the reduction was minor and insignificant when compared to the untreated group. This minor reduction can be attributed to the fact that minoxidil has been shown to inhibit 5AR expression in keratinocyte cell lines^43^. LSLE was able to decrease the concentration of 5AR in the skin homogenate which could account for the rapid hair growth observed in the mice after 21 days and may be the reason why members from the same family of LSLE have been used for centuries as hair tonics^44^. After stopping the treatment, the LSLE remained for 4 weeks as the concentration of 5AR was still below that of group II despite the administration of Sustanon.

AGA has commonly been characterized by a marked decline in the supply of blood, oxygen, and nutrients to the hair follicles. It is worth mentioning that DHT binds to androgen receptors on the hair follicles causing apoptosis of the dermal papilla (DP cells), disrupting the proliferation of keratinocytes and impairing the action of growth factors^45^. We herein evaluated the levels of three fundamental growth factors (VEGF-CTGF-FGF) for hair growth amongst different treatment groups. These factors play a key role in promoting vascularization, fostering angiogenesis, and thereby enriching the follicles with the nutrients and oxygen required for hair growth^46–48^.

Vascular endothelial growth factor (VEGF) is an autocrine growth factor that acts by directly interacting with hair DP cells^49,50^, resulting in increased follicle diameter^51^. It is a biological marker for hair follicle stimulation and growth^51^. Minoxidil, an FDA-approved alopecia treatment, has been recognized for its ability to increase the expression of VEGF in hair DP cells, which stimulates hair growth^52^. Nonetheless, the therapeutic effects of topical Minoxidil 5% can be reversed or reduced following treatment discontinuation^53^.

In the current study, the *LSLE*—Sustanon group demonstrated the highest levels of VEGF (5-folds). This increase was significantly higher than that observed with Minoxidil 5%—Sustanon group (2.5-folds) and remained so even after treatment discontinuation. As such, our findings suggest that *LSLE* possess a robust potential for the treatment of androgenic alopecia and produces a more prolonged therapeutic effect than Minoxidil.

The connective tissue growth factor (CTGF, or CCN2) is a matricellular protein that actuates other Extracellular Matrix (ECM)- proteins such as VEGF, transforming growth factor-β (TGF-β), integrin receptors, fibronectin, type-I collagen, and mucins^54–56^. Accordingly, CTGF serves multiple biological functions such as angiogenesis, cell growth and tissue repair. Animal studies established a strong association between deficiency in the CTGF gene product and development of alopecia in transgenic mice^57^. The knockout CTGF mice also demonstrated deterioration in the angiogenesis process^56^.

In this study, Sustanon-only group (where alopecia was induced without any treatments) demonstrated the lowest CTGF level which lies in accordance with previous studies. Meanwhile, the CTGF level of the *LSLE*—Sustanon group surpassed that of Minoxidil 5%—Sustanon group even after discontinuation of treatment. The exceedingly high level of CTGF observed following *LSLE* treatment, as well as the persistence of such an elevated CTGF level after treatment cessation- further asserts its effectiveness for use against androgenic alopecia.

The connective tissue growth factor (FGF) usually exists among other growth factors like TGF-β, VEGF, and epidermal growth factor in platelets of blood plasma. The release of these growth factors is crucial to bolster the differentiation of stem cells into hair follicles by upregulating the transcription of β-catenin^58^. These factors also possess an anti-apoptotic activity by stimulating the anti-apoptotic signaling pathways including Bcl-2 and Akt, hence, interrupting the catagen phase while prolonging the anagen phase and promoting hair growth^45^. Our study showed that FGF levels of the *LSLE*-Sustanon group exceeded that of Minoxidil 5%-Sustanon group even after treatment discontinuation which again reinforces the beneficial therapeutic effects of *LSLE* in androgenic alopecia.

Despite being the approved drug for AGA treatment, patients’ compliance to Minoxidil is hardly achieved^59^. This can be attributed to a variety of reasons; the relatively high cost of the drug^60^, the adverse effects that follow its application such as pruritus, rash, dandruff, desquamation, allergic contact dermatitis^60,61^ and systemic hypotension^62^, in addition to the long time it takes for the results to be apparent whereby the patient may wait for up to 6 months to obtain a noticeable effect^53^. In a number of reputable papers that were published, it was stated that topical application of 5% minoxidil might have caused non-arteritic anterior ischemic optic neuropathy that was cured after the treatment stopped^63^. Consequently, it is important to search for better alternatives.

It is noteworthy to mention that compared to the approved drug Minoxidil, *LSLE* extract possessed higher inhibitory activity on 5α-reductase and resulted in significantly higher levels of the examined growth factors (VEGF, CTGF and FGF) even after treatment cessation. The immense increase in growth factors following LSLE application secures the supply of oxygen, blood and nutrients to the scalp and lowers the rate of alopecia.

In conclusion, the present study gives an insight about the promising hair-growth and anti-androgenic effects of LSLE. Further research on the anti-apoptotic activity, as well as the systemic absorption of LSLE may be needed to unravel the underlying molecular mechanisms and ensure its safety*.*

## Supplementary Information

Supplementary Information.

## Data Availability

The datasets analyzed during the current study will be available from the corresponding author on reasonable request.
