# Supplementary material for: The medicinal activity of lyophilized aqueous seed extract of Lepidium sativum L. in an androgenic alopecia model
Source: Sci Rep. 2023 May 11;13:7676. doi: 10.1038/s41598-023-33988-1 (PMC10175567; doi:10.1038/s41598-023-33988-1)
Supplement: Supplementary file 1 — Supplementary Information. [file 41598_2023_33988_MOESM1_ESM.docx]

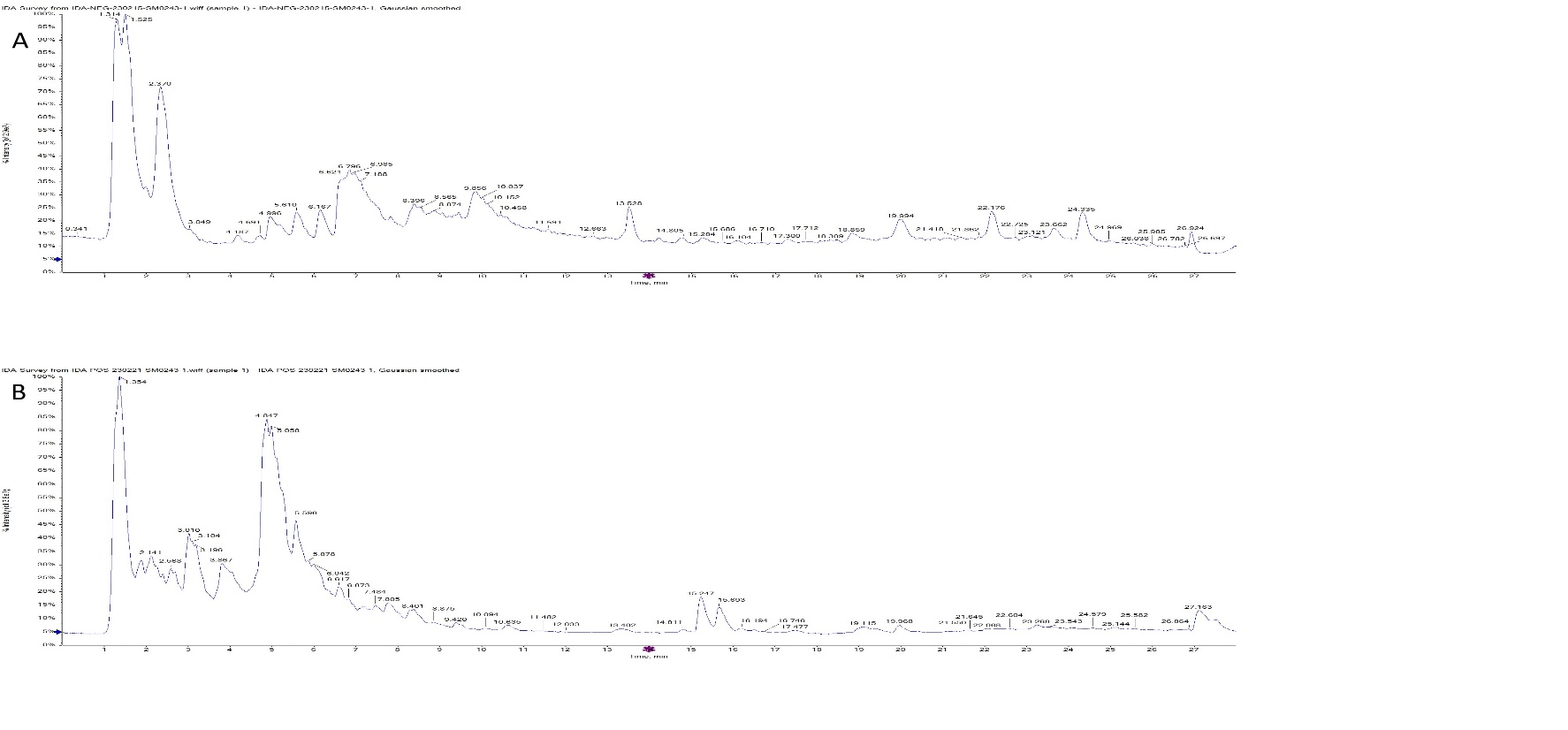


Figure S1: Total ion chromatogram by LC-MS/MS of LSLE labeled with the tentatively identified active metabolites in (A) negative ion mode, and (B) positive ion mode
